# Global Maps of Avian Leukosis Viruses: Research Trends and Themes Based on Networking

**DOI:** 10.3390/vetsci10010016

**Published:** 2022-12-26

**Authors:** Gul Zaib, Xuming Hu, Hengmi Cui

**Affiliations:** 1 College of Veterinary Medicine, Yangzhou University, Yangzhou 225009, China; 2 Institute of Epigenetics and Epigenomics, College of Animal Science and Technology, Yangzhou University, Yangzhou 225009, China

**Keywords:** avian leukosis viruses (ALVs), bibliometric analysis, bibliometric-ALV, research proliferation, VOSviewer, Biblioshiny, network analysis

## Abstract

**Simple Summary:**

Avian leukosis viruses (ALVs) are important veterinary viruses. Given the medicinal and economic significance of ALV infections in poultry, we explored and compiled data from the Web of Science (Core Collection) database. According to scientific data, there are limited bibliometric studies on ALV research. We aimed to find research trends and themes, the influential journals, authors, countries, institutions, and worldwide collaborative networking of countries on ALV research. Recent developments in networking science and bibliometric tools have paved the way to visualize and map scientific outputs. We utilized the bibliometric tools VOSviewer and Biblioshiny software to construct and map research trends and themes for Avian Leukosis Viruses. Our findings revealed that Chinese and American research institutions produced the majority of papers during the study time period. The Journal of Virology and Avian Diseases appeared as the preferable journal/source for publications. Apart from the avian leukosis virus and the ALV-J, the important keywords mentioned were avian leukosis virus subgroup j, chicken, and retrovirus. The bibliometric analysis revealed substantial findings on ALV research, with a strong research response from the USA and China.

**Abstract:**

Avian leukosis virus (ALV) has a tremendous adverse impact on the poultry industry. Since its discovery, research on different aspects of ALV have been published. Due to the vast academic emphasis and economic importance of the ALV infection in poultry worldwide, this bibliometric analysis explored the scientific output associated with ALV utilizing the Web of Science (Core Collection) database. The relevant data were collected using the search query “AVIAN LEUKOSIS VIRUS”, further refined by document types (article, book chapter, and proceedings paper). Finally, 1060 items with full records were imported in Plaintext and tab-delimited formats. The data analysis was carried out using MS Excel, VOS viewer, and R (Biblioshiny) software. Chinese and American research institutions produced the majority of papers during study time period. The Journal of Virology and Avian Diseases appeared as the favorite journal/source for publications. Apart from the avian leukosis virus and the ALV-J, the important keywords mentioned included avian leukosis virus subgroup j, chicken, and retrovirus. The analysis revealed substantial findings on ALV research, with a strong research response from the USA and China.

## 1. Introduction

Avian leukosis viruses (ALVs) are important veterinary viruses economically and medically and are classified based on sequence similarities. These belong to the alpharetrovirus genus of the subfamily orthoretrovirinae in Retroviridae [1,2,3]. Based on their endogenization and transmission mode, ALVs are categorized into endogenous and exogenous avian retroviruses. All oncogenic ALVs fall into the exogenous category of ALV [3] and are causative of severe commercial losses in the poultry industry [4,5]. In chicken, the exogenous ALVs include subgroups A, B, C, D, J, and K, while subgroup E belongs to endogenous ALV [1,5,6,7]. The research on avian retroviruses is not new in the scientific world, as it dates back more than a century [4,8,9], and still, scientists are exploring its enigma. Until now, no effective vaccine has been available for ALV infection [10]. 

Bibliometric studies pave our understanding to determine the scientific impact and research trends. Several indices (indexes), including co-occurrence, citations, and keywords, are widely used in scientometrics to highlight research productivity, collaboration networks, and trends. Scientists use different algorithms and methods to decipher broad dimensions of bibliometric studies [11,12,13]. The bibliometric metadata of scientific publications has been mathematically used for the quantitive analysis of a specific topic’s yearly or total research publications. This method helps to determine the quality of studies, analyze the main areas of research, and also helps to predict future research directions [14]. The network science and bibliometric studies combine to make research more interactive, allowing users to visualize worldwide scientific activity and create maps—the current study utilized bibliometric and networking tools to decipher the ALV research. Our aim was to find out research trends and themes, the influential journals, authors, countries, institutions, and worldwide collaborative networking of countries on the ALV research. According to scientific data, there are limited bibliometric studies on ALV research.

Furthermore, the co-author network analysis helps to explore and quantify research and development collaboration among authors, institutes, and countries [15]. In these graphical visualizations of the co-author network, the authors are nodes interconnected through co-authored scientific publications, which are the edges between these nodes. The level of betweenness density of each author node specifies their connectivity [14].

## 2. Materials and Methods

### 2.1. Data Mining

We collected published datasets indexed in Thompson’s Institute for Scientific Information Web of Science (WOS core collection) with a timeframe of 1999–2022. We searched with the keyword “AVIAN LEUKOSIS VIRUS”. The full citation information, including the authors’ names, organizational affiliations, the publication year, and the sum of citations, was chronicled as a bibliographical corpus in Plaintext and tab-delimited formats [16].

### 2.2. The Inclusion and Exclusion Criteria of Data

The inclusion criteria were: a clear correlation with ALV and a focus on avian subjects. We included pertinent avian leukosis virus articles; review papers; books; editorials; letters; and conference proceedings. Moreover, publications in other languages available on WOS (Core Collection) were also included in our research. Our search did not include studies in other databases, such as Scopus and PubMed.

### 2.3. Bibliometric Analysis

Bibliometric analysis is a new approach to identifying current trends in the field of scientific publications. Over the course of a decade, this study examined publication patterns (top 10 prolific countries and journals), publish themes and trends (top keywords for avian leukosis virus, research focus, and data), scientist contributions, global collaborations, and co-citing references in avian leukosis virus (ALV) research, where “Total Link Strength (TLS)” is an essential feature of bibliometric analysis, as it infers the connectivity among various terms that occur together. The impact factors (IFs) of the journals/sources were manually added from the journal citation report (JCR) (Clarivate Analytics, Philadelphia, United States, available from https://jcr.clarivate.com/) accessed on 26 August 2022 [17]. The VOSviewer software version 1.6.17 [18], MS Excel, and RStudio (Biblioshiny) were utilized to analyze and visualize our dataset.

## 3. Results

Overall, 1060 studies available on the WOS (Core Collection) were included in the recent study with a timespan of 1999–2022; the publication type, annual publication numbers, and total of citations were also investigated. The total citations of these articles were 22,117, and the average sum of citations per article was 20.87. The h-index of these publications was 67. This data set comprises original articles (966; 91.13%) and review articles (64; 6.03%). Most of the publications were in English (1049; 98.96%), followed by Hungarian (3; 0.28%), as shown in Table 1. A sum of 54 countries scientifically contributed to the ALV literature globally. These findings reveal that the USA contributed significantly (444; 41.88), followed by China (419; 39.52%) and England (76; 7.17%). The dataset reveals that Virology (314) and Veterinary Sciences (305) are the chief research areas in the ALV research.

### 3.1. Persuasive Research Journal/Sources

**Figure 1:**
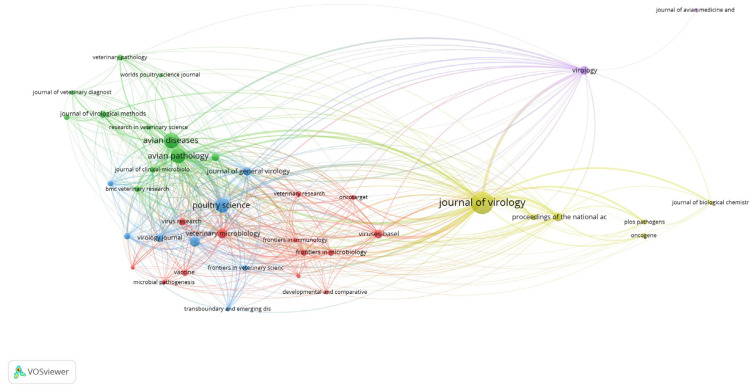
The most influential journals in the avian leukosis virus research. The node size represents the total articles published.

Table 2 shows the ten leading research journals/sources that published scientific literature on ALV research. The *Journal of Virology* (impact factor 4.50) appeared as a leading journal/source (140 publications and 4574 citations), followed by *Avian Diseases* (66 publications and 1180 citations) and *Avian Pathology* (60 publications and 1307 citations) (Figure 1).

### 3.2. Scientifically Contributing Countries and Organizations in Terms of ALV Research

**Figure 2:**
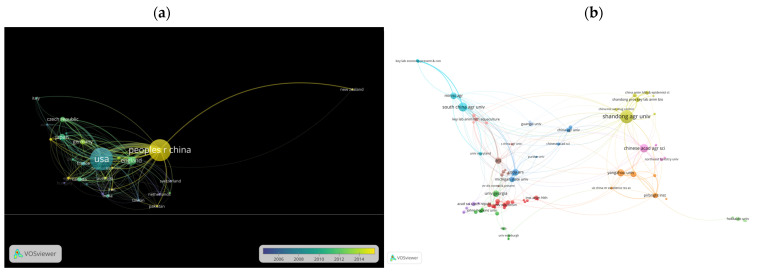
Influence visualization of the most influential countries and organizations in avian leukosis virus research. (**a**) Overlay visualization of prolific countries in the field of ALV research. The color difference describes the research contribution over time. (**b**) Network visualization of the most persuasive organizations in the field of ALV research. The size of a node represents the total productivity of articles, and the distance signifies how these are related to each other.

Table 3 reveals the ten prominent countries and institutions producing ALV publications globally. Only three countries produced more than 50 publications. According to the study, behind the United States of America (USA), which had 443 articles, China came in second with 419 publications (citations 4997, TLS 86), and England followed in third with 76 publications (citations 1981, TLS 69). The findings reveal that the USA had contributed more to scientific ALV-related publications (Figure 2a). Still, the overlay visualization indicates that during the past decade, China has emerged as the knowledge hub for ALV research (Figure 2a). 

Of the top 10 research institutions (Table 3), Shandong Agricultural University (Figure 2b) produced 162 publications and 2092 citations, followed by South China Agricultural University (73 publications and 774 citations) and the Chinese Academy of Agricultural Sciences (64 publications and 879 citations).

### 3.3. Most Cited Documents/Articles

The ALV research is relatively nascent compared with the remaining members of the family Retroviridae; therefore, many aspects of ALV research overlap or depend on the retroviral research dimensions, including DNA integration and endogenous retroviruses. Table 4 describes the top-ten highly cited articles on ALV research. The most cited article, entitled “*Retroviral DNA integration: ASLV, HIV, and MLV show distinct target site preferences*” by “Mitchell RS” published in *Plos Biology* achieved the highest number of citations (703). The *Journal of Virology*’s published literature made up two of the top ten most cited papers. Over 200 citations were received by half of the articles (Table 4). 

### 3.4. The Authorship Analysis

**Figure 3:**
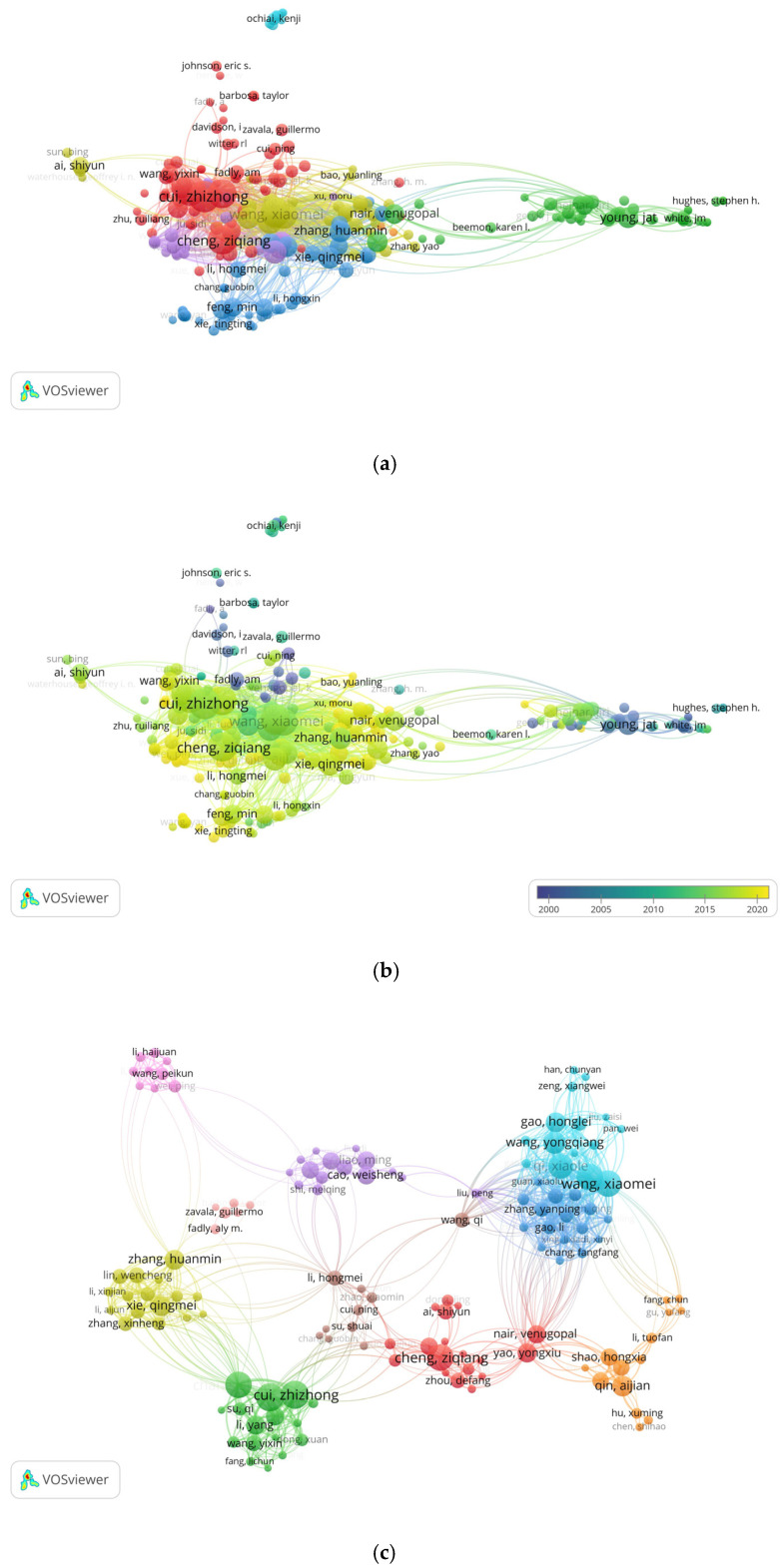
The authorship analysis of authors/researchers/scientists. Each node represents an author, the node’s size shows the author’s count of publications, and each line/edge connecting two authors/researchers indicates the betweenness among researchers. (**a**) The network-visualization map of most cited researchers/authors in the ALV literature. (**b**) Overlay visualization map of the most cited researcher/authors in the ALV research. (**c**) Co-authorship collaboration among researchers. Every node symbolizes a researcher, and the line’s thickness denotes the frequency of co-authorship collaboration amongst the authors.

The citation analysis of scientists/authors was based on the publication count and citations. Out of 3369 authors, only 221 met the default criterion and participated in the scientific ALV research literature worldwide. These authors are in seven clusters, with 12,174 links and 76,335 TLS (Figure 3a,b). Young JAT appeared as a highly cited author, with 1889 citations and 28 documents (Table 5).

The co-authorship network is as much a network depicting academic society as it depicts the structure of our knowledge [15]. Studies on co-authorship networks of authors have gathered the attention of many scholars, not just because of their strong descriptive and synthetic power to define the evolution of scientific research communities but also because social networks play a substantial role in the generation of knowledge and provide a vision of cooperation patterns between individuals [15,19,20]. The analysis reveals the social structure of the networks of the authors/researchers and their connections. This manuscript also reviews the co-authorship network analysis of authors/scientists/researchers (Figure 3c) in ALV research [19]. Current findings reveal Cui Zhizhong as the most co-authored author/researcher (50 articles, 824 total citations, and 226 TLS), followed by Zhao Peng and Wang Xiaomei (Table 5). These findings show that Chinese authors have stronger scientific collaborations in the field of ALV research and the moving of the research hub towards China, which puts more responsibility on Chinese researchers to contribute significantly to the scientific community.

### 3.5. ALV Research Trends and Themes Based on the Co-Occurrence of Author-Provided Keywords

**Figure 4:**
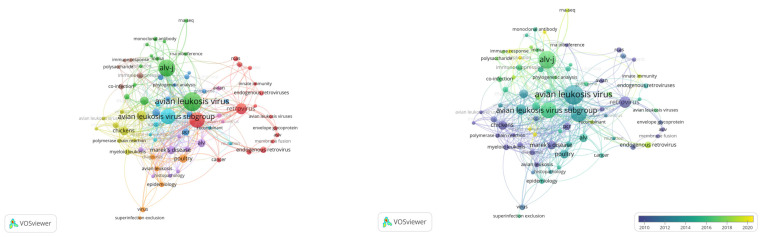
Authors’ keyword analysis for latest research trends on Avian Leukosis Virus literature from VOS viewer software.

Figure 4 presents the research trends based on the authors’ keyword analysis of the avian leukosis virus literature from VOS viewer software. The default occurrence was selected; hence, out of 1906 author-provided keywords, 76 met the criterion, comprising nine clusters with 395 links and 636 TLS. The node size shows the sum of occurrence and TLS by other items/keywords. The top five author-provided keywords were avian leukosis virus, alv-j, avian leukosis virus subgroup j, chicken, and retrovirus, which occurred 94, 77, 67, 65, and 36 times, respectively (Table 6). Furthermore, “endogenous retrovirus”, “immune response”, “innate immunity”, and “replication” appeared as the latest hotspots in the ALV research themes.

### 3.6. Three-Factor Analysis

**Figure 5:**
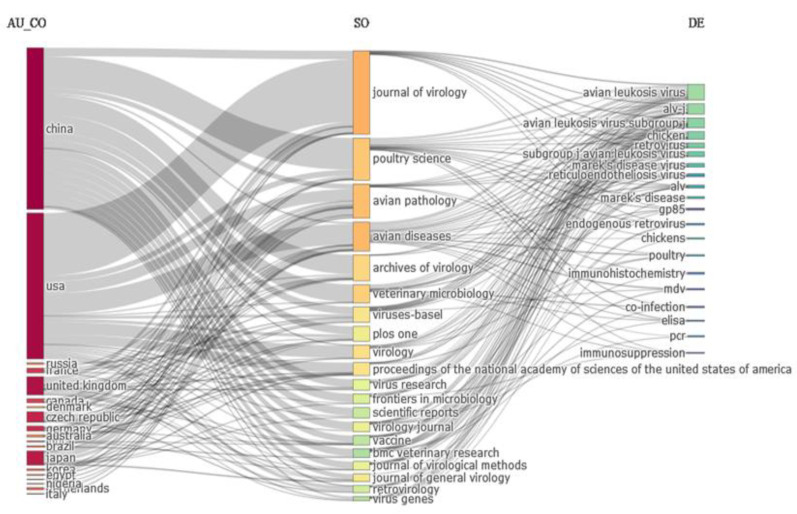
Sankey diagram of three-factor analysis, indicating the association and connectivity between author’s countries (left side), journal/sources (middle), and author-provided keywords (right side).

The three-factor analysis of the association between authors’ countries, journals, and institutions/organizations is mentioned in Figure 5, where the author’s country is shown on the left, journals/sources in the middle, and author-provided keywords on the right side. It shows that the six journals/sources (*Journal of Virology, Poultry Science, Avian Diseases, Avian Pathology, Archives of Virology, and Veterinary Microbiology*) that published the ALV literature with the author-provided keywords (avian leukosis virus, avian leukosis virus subgroup J, ALV-J, subgroup j avian leukosis virus) have a strong relationship with leading countries (China, USA, and the UK, respectively). Figure 5 shows that most US scientists prefer to publish their ALV in the *Journal of Virology*, and that *Avian Disease* is the second-most prioritized source. On the other hand, Chinese scientists prefer to publish most of their ALV work in the *Poultry Science, Archives of Virology*, and *Veterinary Microbiology* journals.

### 3.7. Global Collaboration Map on ALV Research

**Figure 6:**
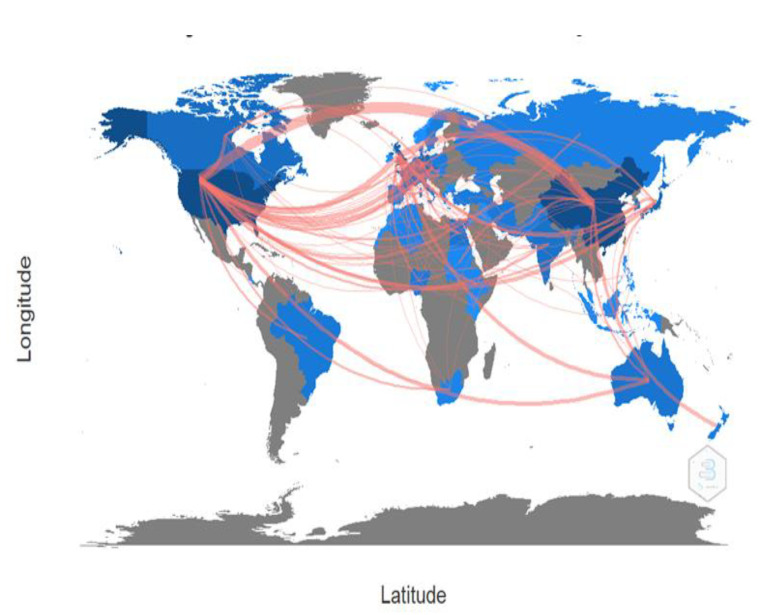
The country-collaboration map among countries and international collaboration in the ALV research.

Figure 6 indicates the country-collaboration map on the ALV research literature worldwide. The USA, the UK, and China are the top collaborating countries, followed by the Czech Republic and Germany. The main collaborating countries with the USA are Australia, Austria, Brazil, Canada, Costa Rica, Czech Republic, Egypt, Germany, Israel, Lebanon, Russia, and United Kingdom. However, the UK has been actively collaborating with Australia, Egypt, Ethiopia, Germany, Iraq, Japan, Korea, and Nigeria. Moreover, China has collaborated significantly with the USA, United Kingdom, New Zealand, Pakistan, Sudan, Canada, and Egypt.

## 4. Discussion

Recently, bibliometric investigations have been utilized more to analyze the scientific research trends and progress in numerous scientific and research domains. The current data analysis represents different dimensions of ALV research, including the leading countries, organizations, and sources/journals producing the literature on ALV. Of these countries, the USA is the leading country in ALV research, closely followed by China. However, during the last decade, China has appeared as the new scientific hub for ALV research. This puts a significant responsibility on Chinese policymakers and researchers to fund more meaningful and citable research [21]. The current findings align with the findings of Al-Jabi and Farooq [22,23], indicating English as the de-facto language, which is also in line with Wiethoelter, Morel, and Yi [24,25,26].

The data analysis regarding authorship and collaborative research patterns reveals that most publications have collaboration ties of authors [15,25,27]. This collaborative network is also essential for health innovation due to its application in interdisciplinary research [19]. The authors from China were the most significant contributors to the topic, which does not coincide with Albuquerque and others [28,29], where US researchers were the most frequent contributors to the studies conducted on Zika. Moreover, the articles from the USA were the most cited, followed by China. Shandong Agricultural University was the most cited institution, followed by South China Agricultural University [30]. Considering the country-wise organizations contributing to the theme, it is not astounding that Chinese institutes ranked on top in the ten lading organizations list, followed by the USA organizations. The bibliometric dataset unveiled the most productive journal on ALV research. According to our findings, the *Journal of Virology* has published more on the ALV topic. The present results coincide with the previous study of Zaib et al. [11], where the *Journal of Virology* appeared as the most influential journal. These findings contradict the results of Koo [31], who reported that the *Journal of Alternative and Complementary Medicine* contributed the greatest number of articles on aroma therapy. These findings are also not in line with the study conducted on Campylobacter [32]. The country-collaboration map centering a significant count of the literature published as the result of positive collaborative research between countries, such as the USA and China, agrees with Hossain’s bibliometric work [32]. The USA actively participated in collaborative research with the UK and Canada, which matches the previous study [23].

Early studies mainly concentrated on the diagnosis, signs, and subtypes of ALV, but later they prioritized ALV-J more due to the economic losses in the poultry industry. There is inadequate data available for the treatment and vaccine development against ALV infection among poultry flocks. Hence, scientists started to study the immune response, replication, and innate immunity to curb the infection in the host.

The current study has some limitations, such as avian leukosis virus research data collection being limited to the WOS (Core Collection) publication index, whereas other databases such as PubMed, EMBASE, Google Scholar, Dimension, and Scopus were not included. Moreover, gender analysis was not performed to study females’ global contribution to the ALV research. Future work in these domains may provide a thorough comparative and cumulative aspect of the ALV literature available on these databases.

## 5. Conclusions

Bibliometric analyses are now being undertaken in a variety of scientific domains, but the current study is believed to be the foremost for ALV research. In short, the bibliometric analyses were carried out to better understand and quantify the global research productivity for ALV. China and the United States appear to collaborate with one another, and the rest of the globe, the most, and have the highest research productivities. Regarding publishing output, the Chinese Academy of Sciences (CAS), South China Agricultural University, and Shandong Agricultural University hold the top three spots among research institutions. Moreover, the analysis highlighted that “endogenous retrovirus”, “immune response”, “innate immunity”, and “replication” emerged as the dominant research trends and themes. The preferable journals for publication are *the Journal of Virology* and *Avian Diseases*. In general, the current study provides a comprehensive overview of the global ALV research perspective, and therefore can serve as the basis for future research in this field.

## Figures and Tables

**Table 1:** WOS (Core Collection).

Parameters		Sum	Frequency %
Document Type	Article	966	91.132
	Review Article	64	6.038
	Meeting Abstract	14	1.321
	Proceeding Papers	9	0.849
	Book Chapters	7	0.660
Languages	English	1049	98.962
	Hungarian	3	0.283
	Polish	3	0.283
	German	2	0.189
	Dutch	1	0.094
	Portuguese	1	0.094
	Turkish	1	0.094
Research Areas	Virology	314	29.623
	Veterinary Sciences	305	28.774
	Biochemistry Molecular Biology	91	8.585
	Agriculture	89	8.396
	Microbiology	87	8.208
	Biotechnology Applied Microbiology	75	7.075
	Science Technology Other Topics	63	5.943
	Immunology	62	5.849
	Genetics Heredity	56	5.283
	Cell Biology	41	3.868

**Table 2:** The most influential journals/sources in the avian leukosis virus research.

Ranks	Journals/Source	IF	TP	TC	TLS
1	*Journal of Virology*	4.50	140	4574	929
2	*Avian Diseases*	1.73	66	1180	740
3	*Avian Pathology*	3.37	60	1307	807
4	*Poultry Science*	3.19	57	706	581
5	*Veterinary Microbiology*	3.29	27	341	417
6	*Archives of Virology*	2.57	26	337	337
7	*Virology*	3.61	22	554	278
8	*Proceedings of National Academy of Sciences*	11.20	21	1055	198
9	*Viruses-Basel*	4.91	21	167	257
10	*Journal of General Virology*	3.37	19	430	384

IF = impact factor of journals/sources; TP = total production; TC = total citations; TLS = total link strength.

**Table 3:** Ten prominent and most prolific countries and research institutions in ALV research.

Parameters		TP	TC	TLS
Countries	USA	443	13758	119
	Peoples R China	419	4997	86
	England	76	1981	69
	Japan	45	431	16
	Germany	31	1335	39
	Czech Republic	31	384	14
	Canada	22	338	16
	France	19	367	19
	India	14	125	5
	Scotland	13	243	26
Research Institutions	Shandong Agricultural University	162	2092	119
	South China Agricultural University	73	774	142
	Chinese Academy of Agricultural Sciences	64	879	50
	Yangzhou University	48	460	49
	United States Department of Agriculture USDA ARS	42	1062	53
	Ministry of Agriculture	41	618	90
	University of Georgia	36	569	17
	Michigan State University	28	440	37
	Pirbright Institute	25	173	43
	University of Wisconsin	23	812	18

TP = total production, TC = total citations, TLS = total link strength.

**Table 4:** Globally ten leading and highly cited articles in the field of avian leukosis virus.

	Title	Authors	Journals	IF	PY	TC	TC/Y	DOI
1	*Retroviral DNA integration: ASLV, HIV, and MLV show distinct target site preferences*	Mitchell, R.S.; Beitzel, B.F.; Schroder, A.R.W.; Shinn, P.; Chen, H.M.; Berry, C.C.; Ecker, J.R.; Bushman, F.D.	*Plos Biology*	9.59	2004	703	37	10.1371/journal.pbio.0020234
2	*The evolution, distribution and diversity of endogenous retroviruses*	Gifford, R.; Tristem, M.	*Virus Genes*	2.27	2003	264	13.2	10.1023/A:1024455415443
3	*Retroviral entry mediated by receptor priming and low pH triggering of an envelope glycoprotein*	Mothes, W.; Boerger, A.L.; Narayan, S.; Cunningham, J.M.; Young, J.A.T.	*Cell*	41.58	2000	249	10.83	10.1016/S0092-8674(00)00170-7
4	*The discovery of endogenous retroviruses*	Weiss, Robin A.	*Retrovirology*	4.18	2006	223	13.12	10.1186/1742-4690-3-67
5	*Viral Nucleic Acids in Live-Attenuated Vaccines: Detection of Minority Variants and an Adventitious Virus*	Victoria, Joseph G.; Wang, Chunlin; Jones, Morris S.; Jaing, Crystal; McLoughlin, Kevin; Gardner, Shea; Delwart, Eric L.	*Journal of Virology*	4.50	2010	220	16.92	10.1128/JVI.02690-09
6	*The long view: 40 years of avian leukosis research*	Payne, L.N.; Nair, V.	*Avian Pathology*	3.37	2012	198	18	10.1080/03079457.2011.646237
7	*A review of the development of chicken lines to resolve genes determining resistance to diseases*	Bacon, L.D.; Hunt, H.D.; Cheng, H.H.	*Poultry Science*	3.19	2000	168	7.3	10.1093/ps/79.8.1082
8	*Symmetrical base preferences surrounding HIV-1 and avian sarcoma/leukosis virus but not murine leukemia virus integration sites*	Holman, A.G.; Coffin, J.M.	*Proceedings of the National Academy of Sciences*	11.20	2005	135	7.5	10.1073/pnas.0501646102
9	*Development of a flexible and specific gene delivery system for production of murine tumor models*	Fisher, G.H.; Orsulic, S.; Holland, E.; Hively, W.P.; Li, Y.; Lewis, B.C.; Williams, B.O.; Varmus, H.E.	*Oncogene*	8.75	1999	124	5.17	10.1038/sj.onc.1203087

IF = impact factor of journals/sources; PY = publication year; TC = total citations; TC/Y = total citations per year.

**Table 5:** Top ten cited authors in the ALV research.

Authors		Doc	TC	TLS
The most cited author in the ALV	Young Jat	28	1899	1506
	Callaway, Edward M	5	1247	23
	Cui Zhizhong	50	824	4111
	Li Y	5	670	19
	Wang Xiaomei	44	644	4190
	Gao Yulong	43	644	4185
	Qi Xiaole	39	618	3879
	Zhao Peng	49	573	3472
	Gao Honglei	26	542	2864
	Chang Shuang	43	502	3228
Co-authorship analysis of scientists	Cui Zhizhong	50	824	226
	Zhao Peng	49	573	240
	Wang Xiaomei	44	644	379
	Gao Yulong	43	644	379
	Chang Shuang	43	502	229
	Cheng Ziqiang	43	479	116
	Qi Xiole	39	618	359
	Wang Yongqiang	33	599	269
	Qin Aijian	29	273	99
	Gao Honglei	26	542	186

Doc = total articles/documents published by scientists, TC = total citations, TLS = total link strength.

**Table 6:** Author-provided keyword analysis on Avian Leukosis Virus literature.

No.	Keywords	Occurrence	Total Link Strength
1	avian leukosis virus	94	101
2	alv-j	77	71
3	avian leukosis virus subgroup j	67	46
4	Chicken	65	96
5	Retrovirus	36	40
6	Subgroup j avian leukosis virus	29	30
7	Marek’s disease virus	22	30
8	Marek’s disease	21	42
9	Reticuloendotheliosis virus	18	27
10	Endogenous retrovirus	13	12

## Data Availability

The dataset in the current study comes from the Web of Science (core collection) and is available on its web page. Thomson Reuters does not allow us to make the data freely available. Readers can contact Thomson Reuters to obtain the data (http://thomsonreuters.com/thomson-reuters-web-of-science/, accessed on 1 November 2022).
